# ‘*It was called a grab bag and nobody wanted to grab them’*: Teachers’ perceptions of school lunches during the COVID-19 pandemic – a regional case study

**DOI:** 10.1177/22799036231193071

**Published:** 2023-08-21

**Authors:** SMM Zaremba, WB Cook, AS Anderson

**Affiliations:** 1 Division of Population Health & Genomics, Ninewells Hospital & Medical School, University of Dundee, Dundee, UK; 2 NHS Tayside, Dundee, UK

**Keywords:** COVID-19, school meals, food provision, Scotland

## Abstract

**Background:**

The COVID-19 pandemic brought changes to primary school lunches leading to concerns over nutritional quality and uptake of lunches by vulnerable children. Regional data from Tayside, Scotland, showed that only 55% of children who were eligible for free school meals took these (normal uptake pre-pandemic was 66%). The current work aimed to identify teachers’ perceptions of meal provisioning in primary schools during the first year of the COVID-19 pandemic.

**Design and methods:**

A cross-sectional online survey was carried out among primary school teachers across Tayside, Scotland. Using an online survey (21 questions combining multiple choice formats and open text) and interviews, primary school teachers shared their views on food quality, quantity, meal choices and factors influencing uptake of primary school lunches. Interviews were recorded, transcribed and thematically analysed with respect to factors influencing consumption.

**Results:**

The survey was completed by 41 teachers and 8 participated in a follow up interview. Around one-third (29%) of primary school teachers believed the quality of lunches had decreased and cited poor appearance of food, use of takeaway containers and food wastage. The lunch format was viewed negatively principally relating to the substitution of hot lunches with cold sandwiches, portion sizes, choice and perceived value for money. Concerns were expressed about acceptability and how far the meals contributed to food security.

**Conclusions:**

Further work on food provisioning is needed in order to identify ways to provide a nutritional safety net for vulnerable children.

## Introduction

The importance of school lunch and the need for nutritional standards has long been recognised as a key factor in the nutritional well-being of children.^1^ In a wider context, the Scottish Government^2^ has also stated that ‘*food in school matters – both what children and young people eat and what they learn about. It impacts upon their health, on their education, and on the environment and economy*’. In turn, it has also been demonstrated that the school environment impacts on food knowledge, well-being and food intake.^3^

In Scotland, statutory nutrient standards for school lunches have been designed and implemented to address the challenge of supplying a significant proportion of daily nutrient requirements whilst avoiding excess caloric intake and thus contributing to the increasing rate of childhood obesity.^4,5^ However, the COVID-19 pandemic brought school lunches sharply into focus due to replacement meal strategies, new menus with altered food choices and delivery criterion. These changes were brought about to minimise the risk of COVID-19 spread and to facilitate a daily meal service at a time when resources, including staff and ingredients, were reduced.

The importance of school lunches during the COVID-19 pandemic has been highlighted by children, parents, politicians, media, nutritionists and paediatricians.^6,7^ Health concerns relate to increased risk of both under-nutrition and overweight. COVID-19 protection procedures have impacted on children’s psychological well-being, food choices, snacking and sedentary time as well as decreasing levels of physical activity and it is thought these responses are greatest in children with excess weight.^7^ During the same period, data from Public Health Scotland^8^ reported a 6.8% increase in the number of children at risk of overweight and obesity, with the largest increases (8.5%) in those living in areas of high deprivation.

During the first year of the COVID-19 pandemic, there were clear indicators of increasing food poverty throughout the country and figures collected in July 2020 suggested an increase of 53,000 children and young people were in receipt of free school meals (FSM), vouchers or cash since the COVID-19 outbreak began.^9^ In addition, reports indicated that more children were arriving at school hungry.^10^ During the 2020/21 period of the COVID-19 pandemic, the percent of all primary school children across the Tayside region in Scotland taking meals provided by the catering contractor was 39% compared with a normal uptake of 51%. Uptake of free school meals also decreased, with 55% of all primary children who were eligible taking these compared to normal uptake of 66%.^11^

As food poverty increases throughout the UK, vulnerable children who may have limited access to high quality food at home stand to gain most from the provision of a nutrient rich mid-day meal, indeed school lunches are considered to be a ‘nutritional safety net’ for many children. Work by Obesity Action Scotland^12,13^ has highlighted that prior to the COVID-19 pandemic, school meals were far from optimal (both in provision and setting) and, although there have been some improvements in the last 2 years, more work is needed. Furthermore, the latest report by Obesity Action Scotland^14^ which analysed primary school menus from April 2021 to March 2022 showed that progress has remained marginal in many areas and that there have been setbacks in specific areas. New school meal standards^15^ were due to be implemented in Scotland in Autumn 2020 but these were delayed due to the COVID-19 situation. The new standards were implemented in April 2021.

In response to dining room distancing requirements due to COVID-19, the Tayside regional catering contractor supplied a cold foods menu to children during the autumn term (August–October 2020) and transitioned to limited hot food options later in the year and onwards into 2021. The extra workload required to deliver food was described by the contractor as more labour intensive than the normal service. Extra duties included delivering meals to classrooms on food trolleys, cutlery being individually wrapped and clearing up processes in each individual classroom. In addition, dining rooms were adapted for multiple sittings with breaks in-between to maintain designated bubbles.

Whilst working in challenging circumstances, the cold food menus offered a practical solution, but concerns were raised about the nutritional quality, uptake by those in receipt of FSM and acceptance by primary school aged children. It was therefore timely to explore experiences of school food provision during the COVID-19 pandemic.

The aims of the current study were to examine primary school food provision (in terms of food-based standards) across COVID-19 transition periods and to identify perceived issues around school meal delivery criteria, eating environments and factors which may be related to reduced uptake of FSM.

## Design and methods

A cross-sectional online survey was carried out among primary school teachers in Dundee City, Angus and Perth & Kinross (Tayside) local authority areas. The required data was collected from 3rd December 2020 to 23rd March 2021. It was not possible for the research team to enter schools due to COVID-19 restrictions, therefore, to gain insight into meal delivery, eating environments and perceived factors influencing uptake, school teaching staff in Tayside were invited to provide details of experience in schools. Following ethical clearance from the University of Dundee Medical School Research Ethics Committee (Approval Number: 20/132), permission was sought and granted from the three local authorities within the region, namely Dundee City, Angus and Perth & Kinross to contact primary school teachers to participate in an online survey and follow up interviews.

**Figure 1.**
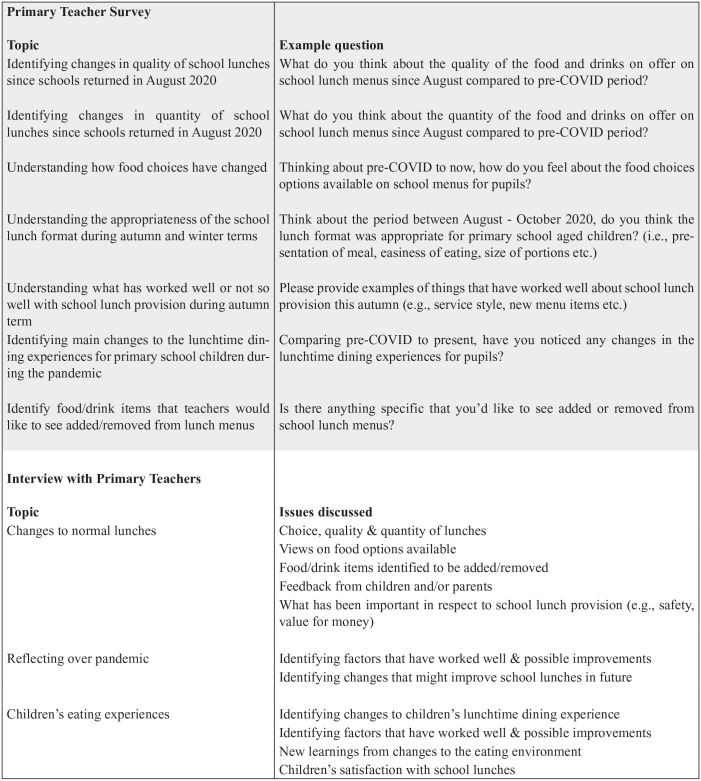
Topics and themes explored in primary school teacher survey and interviews.

The survey was distributed by e-mail to all primary school headteachers or via the research team. Primary school teachers were invited to complete the anonymous survey, with the opportunity to opt into a follow up online interview with a researcher (SZ) to explore factors related to school lunch provision during COVID-19 further. The survey comprised 21 questions combining multiple choice formats and open text. Interviews were recorded, transcribed, and thematically analysed and interpretations were discussed between the research team (AA, WC, SZ) with respect to factors influencing consumption. Figure 1 summarises key themes explored in the online survey and interviews.

## Results

### Responses

Out of a possible 153 primary schools within Tayside (covering the local authorities of Dundee City, Angus and Perth and Kinross) survey responses were received from 41 teachers across 25 schools. Five schools had >40% of children registered for FSM, four schools had 30%–40%, two had 20%–30%, six had 10%–20% and the remainder had <10%.

Eleven primary school teachers showed interest in the follow-up online interview and eight teachers (including two head teachers) completed the interviews during January to March 2021. An even spread of teachers were interviewed according to percent of children registered for FSM within their school. Three schools had 0%–10% children registered for FSM, one school with 10%–20%, two schools with 30%–40% and one school with 40%–50% children registered for FSM. All teachers interviewed had experience of the school-based working environment during the pandemic.

### Food provision and practice

Data from Tayside Contracts (the commercially based local authority contracting organisation responsible for school catering throughout Tayside) provided information on food provision. **Meal choices** across three terms of academic year 2020/21 are detailed in Table 1.

**Table 1.** Menu lunchtime choices across three terms.

	Autumn term	Winter term	Spring term
Tayside contracts primary school lunch offer	Two cold choices	One hot choice or mixed hot/cold second choice	Soup plus two hot choices or third hot/cold option

### Eating environment, meal delivery and factors affecting food intake

#### Location

Two-thirds (*n* = 31) of survey respondents reported awareness of major changes to school food provision in their school during the autumn 2020 term. One of the main observations reported were the location of where lunch was served. Most respondents reported changes to the dining setting (*n* = 35) with respect to social distancing, staff roles and decreased noise levels.

The interview data revealed mixed responses to the location of eating. A range of positive comments suggested that children liked being in the classroom to eat lunch, with effects such as taking time to eat food provided, talking about food, helping to foster good eating habits and opportunities for educational games. On the other hand, respondents reported that the classroom would have remnant foods and smells and children missed out on outdoor play and social dining experiences.

#### Meal delivery

Eight survey respondents commented on packaging waste (takeaway containers, plastic packaging and paper bags). Interview respondents described considerable concern over the packaging that had been utilised to deliver lunches. Many schools have signed up to Eco-Schools principals and staff were clearly disturbed by the amount of excess plastic packaging.

#### Lunch format

The survey data indicated that during both terms (autumn and winter), most respondents reported that school lunch format was generally appropriate. However, the autumn term format was more likely to be rated *less* appropriate.

The interview data suggested that the lunch bag format used during the autumn terms were viewed fairly negatively, principally relating to the substitution of the hot lunch with a cold sandwich type format, portion sizes, choice and perceived value for money. Significant concerns were expressed about whether these offerings were acceptable for children – especially vulnerable children where the school meal was likely to be the only hot food available:

*“It was pretty much take it or leave it. It was called a grab bag and nobody wanted to grab them”.* (Teacher 8, Angus)

There was an overwhelming sense that a hot meal was more likely to be eaten and therefore contribute to food security. The following comments illustrate some of the views expressed:

*“I think the fact that it’s a hot meal, that it’s got a variety of food within it because that might be the only hot meal that they get in that day”* (Teacher 5, Dundee)

*“we were dancing around here when they put a hot option back on the menu”.* (Teacher 8, Angus)

*“Hot lunches started after the October holidays and one of the P7 boys who is from a really poor background – he’s Asian – he actually cheered and we were trying to rush them on to get them out of the class because they’re using the classroom at that point for eating, and he said ‘this is the best lunch I’ve had since this time last year – I want to savour it!’ It was a jacket potato with beans. . . but that was the first hot lunch that was a Muslim lunch, you know a Halal lunch that he could access”.* (Teacher 3, Dundee)

#### Food quantity

Eighteen survey respondents perceived quantity to have deteriorated mainly due to small portion size. In addition, the inability to attain ‘second helpings’ and larger servings due to COVID-19 restrictions on lunch delivery were noted. Four survey respondents reported improved quantities.

This issue was elaborated on in the interview data:

*“the kids are hungry again halfway through the afternoon, whereas before they might be a bit peckish but you definitely notice that they’re sitting and you can see their attention waning.”* (Teacher 2, Angus)

*“I feel that the portions have been a little bit mean and I think that the sandwich option did not fill children up. . . Some of them eat their lunch and they are still hungry. . . Some of the 12-year-olds are the same height as me.”* (Teacher 8, Angus)

#### Food quality

Around one-third of respondents (*n* = 12) believed the quality of lunches had decreased and cited poor visual appearance of food in takeaway containers and expressed concerns around food wastage. A small number (*n* = 5) reported some improvements.

Interview data with teaching staff reflected specific concerns about food items which had been served ‘buffet style’ and were now served as individual portions to reduce risk of COVID-19 contamination. Bread portions were served (even when bread-based mains were served) as slices ‘wrapped in cellophane’ following the removal of a ‘breadbasket’. Similarly, salad bar services were no longer available and individual portions of salad were given as default to all children. Many comments indicated a clear desire that salad bar formats should return.

Few comments were passed about the nutritional quality of the lunches – with more concerns being expressed about serving sufficient food, as illustrated in the following comment:

*“the nutrition standards is not top of the list for me. I’m more interested in them eating. I’d rather that they ate something than nothing.”* (Teacher 8, Angus)

Most survey respondents (*n* = 18) reported that food choices were less appropriate and comments focussed on limited and/or reduced menu choices, although views on this were mixed. For example, some respondents indicated that less choice was better, but others highlighted this reduction as repetitive and/or limiting:

‘*Kids much prefer having only two choices*’. (Teacher 6, Perth & Kinross)

The content of meal choice was questioned:

‘*There are some strange combinations, pizza, bread and potatoes. Overload of carbs’.* (Teacher 10, Dundee)

Teachers also expressed the view that menu choices should be influenced by what foods are popular for children:

‘*The COVID menu should have focussed on the firm favourites and those easy to present e.g., pizza, veggie hotdogs and meatballs. Some days the majority of some choices went in the bin. I understand the need for variation but during this time we need to ensure that children eat’.* (Primary Teacher 3, Dundee)

Interview respondents also highlighted that children need to know and understand what the choices are and thus meet expectations about food:

*‘As I said I think they just need to call it what it is. If it’s sausages just call it sausages don’t call it something fancy ‘cause I’m talking about primary one, two and three here this is where you have the problems, they just cry, they just cry into their food. They sit and cry and it’s not a nice thing to see because you can’t give them anything else apart from salad and eh so I think just simplify it, tell them what it is and kids will either say yay or nay*’. (Teacher 8, Angus)

#### Value for money

Concerns were expressed over perceived value for money and by implication whether the lunch provision actually met the needs of vulnerable children.

As one respondent noted:

*‘parents were saying they weren’t paying. I put information out to encourage school lunches and parents were saying, ‘we aren’t paying for that, we’re not paying £2 odds for a sandwich and an apple that they don’t even like’. You can get a nicer sandwich for £1.50 at eh the shop down the road so I think it’s about quality as well’.* (Teacher 8, Angus)

Interview responses about perceived vulnerability were also noted:

*‘vulnerable children brought in and they were obviously having a cold breakfast, a cold lunch and for the key workers children staying on beyond 4 [pm] was a cold tea. And that became a challenge as the children just didn’t want to eat it. . . the kids we have that are sitting here are the ones who won’t get proper food at home’.* (Teacher 3, Dundee)

## Discussion

Teachers can play a large role in school lunch and have power to act as agents of cultural change in schools.^16^ To our knowledge, this is the first study that has focussed on primary school teachers’ views and perceptions of school lunch provision in Scotland during the first year of the pandemic. Despite sustained meal provision, uptake of school lunches across all school pupils fell dramatically during the first year of the COVID-19 pandemic and this was of particular concern for the most vulnerable children entitled to free school meals. There may be external reasons for this observation, but the current work suggests that reduction in the uptake of school lunches during the first year of the pandemic can be explained, at least in part, due to changes in eating environments, changes in meal format (e.g. cold food bagged lunches), reduced menu choices, diminished quality and quantity of food, anxiety about risk of COVID-19 transfer and concerns over low value for money. The perceived importance of hot food as an acceptable route for encouraging food uptake was notable.

The provision and quality of children’s food are key issues in the amelioration of food poverty and this is recognised by the Scottish Government in their current free school meals policy^17^ and expansion of this scheme^9^ as well as the implementation of mandatory food and nutrient standards.^15^ Given the crisis situation across Scotland during the COVID-19 pandemic and the rapid local authority responses to the consequences in the form of cold lunch provision, it can be considered that a vital service was delivered by local authorities despite the perceived drawbacks of reduced choice and lack of hot lunches. The current work highlights many of the challenges experienced by local authority catering teams aiming to attain high uptake of quality meals by children and young people during the COVID-19 pandemic. The challenges for caterers cannot be underplayed – but planning for future emergency situations needs to move beyond ‘the basics’ of food provision to incorporating aspects of acceptability and uptake of meals of high nutritional value in vulnerable children. Consideration of a minimum standard for meal provision during any future lockdowns and the recognition that some individuals and their families will need additional support due to shielding, availability of transport and other factors is required.

Children need to eat sufficient food of high nutrient density to satisfy hunger and meet energy and nutrient requirements for growth and development.^18,19^ Concerns about adequate food and ‘giving children what they like’ must be balanced with the need to provide overall nutritional adequacy (including vegetables and fruit) and the avoidance of food habits associated with obesity (sweetened drinks and high calorie desserts).^20,21^ Achieving optimal consumption needs to take account of the eating environment, presentation, understanding of menu choices, menu formats (hot/cold options) as well as taste, quantity and quality of food.^18,22^ Feedback from pupils, parents/carers and school staff should also be used as an opportunity to co-produce menus and shape dining environments to reverse the trend of declining meal uptake. In addition, our findings also highlight the importance of finding ways to maintain environmental sustainability within school settings during periods of uncertainty, particularly around plastic waste.

Currently, the cost-of-living crisis is having a crippling effect on families across the UK, with new figures from The Food Foundation reporting that in September 2022 4 million children were living in households experiencing food insecurity, with larger families (≥1 children) more likely to experience food insecurity than those without children.^23^ School meals offer a vital safety net to children and thus emphasises the importance of delivering nutritious and enjoyable hot school food provision, since more households are affected by food insecurity now than during the height of the COVID pandemic. Hence, school meal satisfaction and availability should be at the forefront of local authority agendas, especially if we should be faced with future food provision challenges. Despite a return to ‘normal’ school lunch services across Scotland in 2022, meal uptake (including those eligible for universal free school meals) is still significantly lower than it was in 2016, 68.3%, 82.7%, respectively.^24^ Lack of access to good food and proper nutrition during childhood can have a devastating effect on children’s educational attainment,^25^ physical and mental health^26,27^ and social wellbeing^28^ – all of which have lifelong consequences.

The current work provides an insight to relevant issues by primary school teachers who chose to participate in the study, illustrating perceptions and experiences of school lunches in primary schools in only one Scottish region (Tayside) during the COVID-19 pandemic, which is a limitation of this research. These views may not be representative of all school staff, parents or pupils but provide indicators of areas for catering service providers to explore further. Some of the teachers included in the study expressed an interest in school food. We also acknowledge that lunchtime observations within primary schools would have been desirable to capture a more realistic snapshot of children’s dining experiences, however this was not possible due to COVID-19 restrictions.

## Significance for Public Health

This study highlights the importance of maintaining school lunch nutritional standards and acceptable models of meal provision during periods of uncertainty/increased risk of food insecurity such as lockdown or school holidays. Systematic review level evidence supports a potentially protective effect of universal FSM on body mass index.^29^ This further highlights the importance of increasing FSM up take across Scotland, given the widening in healthy weight by socioeconomic position since 2019.^8^

## Conclusion

Primary school teachers were able to provide us with unique insight into lunchtime routines and behaviours during the first academic year of the COVID-19 pandemic. Rates of school meal uptake fell during the first year of the COVID-19 pandemic within the Tayside region, and our data suggests that changes in eating environments and meal format, reduced menu choices, diminished quality and quantity of food, anxiety about risk of COVID-19 transfer and concerns over low value for money may be linked to the decrease in reported numbers of primary school children eating school lunches, including those eligible for FSMs. Further work on food provisioning is needed in order to identify ways to provide a nutritional safety net for vulnerable children, particularly now in the midst of the cost-of-living crisis.
